# Detection of acute myocarditis using T1 and T2 mapping cardiovascular magnetic resonance: A systematic review and meta‐analysis

**DOI:** 10.1002/acm2.13365

**Published:** 2021-09-04

**Authors:** Zhi Jia, Lihong Wang, Yanqing Jia, Jun Liu, Hong Zhao, Liwei Huo, Binbin Zheng

**Affiliations:** ^1^ Department of Cardiology Tianjin Beichen Hospital Tianjin China

**Keywords:** diagnosis, ECV, mapping, meta‐analysis, myocarditis

## Abstract

**Objectives:**

This study was aimed to systematically review the existing literature and explore more the diagnostic value of T1 and T2 mapping in acute myocarditis.

**Methods:**

Studies were searched from five electronic databases. Sensitivity, specificity, diagnostic odds ratio (DOR), and summary receiver operating characteristic curves (SROC) were calculated to present diagnostic performance. A meta‐regression and subgroup analysis was performed based on validation (endomyocardial biopsy [EMB] vs. clinical criteria).

**Results:**

A total of 10 studies were included, with 400 myocarditis patients and 266 controls. Native T1, T2, and extracellular volume (ECV) values were significantly increased in the myocarditis group. Pooled sensitivities for T1, T2 mapping, and ECV were 0.84 (0.78–0.88), 0.77 (0.69–0.83), and 0.69 (0.50–0.83), respectively. Pooled specificities were 0.86 (0.69–0.95), 0.83 (0.73–0.89), and 0.77 (0.63–0.87), respectively. The DORs were 32 (12–87), 16 (8–30), and 7 (4–14), respectively. The areas under the curve (AUC) of SROC were 0.87 (0.84–0.90), 0.86 (0.82–0.89), and 0.80 (0.76–0.83), respectively. In the meta‐regression and subgroup analysis, significantly lower specificities of T1 and T2 mapping were observed in EMB studies (*p* < 0.01).

**Conclusion:**

The currently available evidence shows that T1 and T2 mapping including ECV alone offer comparably good diagnostic performance for the detection of acute myocarditis. The reason for the observed mismatch with EMB findings should be further investigated.

## 1 INTRODUCTION

Myocarditis is an inflammatory cardiovascular disease that is always blamed for the progression of myocardial injury, sudden cardiac death, and nonischemic dilated cardiomyopathy.^1^, ^2^ Data in biopsy studies have shown that the inflammation had a contribution to 9% of dilated cardiomyopathy.^3^ Furthermore, it is reported that myocarditis accounts for sudden cardiac death in 20%–40% of young adults.^1^ Although an early and accurate diagnosis of myocarditis is necessary to reduce the risk of progression, the diagnosis is still a challenge in modern cardiology because of the variety of clinical representations of myocarditis and undetermined diagnostic criteria.^4^

Cardiovascular magnetic resonance (CMR) imaging has emerged as a reliable non‐invasive diagnostic tool in patients with suspected myocarditis.^5^ The diagnosis of acute myocarditis using CMR is based on the Lake Louise criteria (LLC), which were recently supplemented by quantitative imaging parameters.^6^ These quantitative techniques consist of T1 and T2 mapping including extracellular volume (ECV) fraction, which allows for the accurate quantification of myocardial fibrosis and edema.^7^, ^8^ Both T1 and T2 mapping have been shown to be reliable diagnostic markers for acute myocarditis and offer a more objective assessment of myocardial tissue characterization while overcoming the limitations of semiquantitative approaches.^5^, ^9^ However, their diagnostic performance remains inconsistent across the literature. Although there were several published meta‐analyses combining evidence to validate their diagnostic quality, some limitations existed and biased the results, such as limited data or a mixed patient cohort of acute and chronic myocarditis.^10^, ^11^, ^12^

To improve the clinical practice of CMR for patients with suspected acute myocarditis, this systematic review and meta‐analysis were conducted to explore more the diagnostic value of T1 and T2 mapping in acute myocarditis by synthesizing more available published studies.

## 2 METHODS

This systematic review and meta‐analysis were present in accordance with the guideline of the Preferred Reporting Items for Systematic reviews and Meta‐Analyses statement (PRISMA).^13^

### 2.1 Study selection

Studies were searched from PubMed, Embase, ScienceDirect, Scopus, and Cochrane Library up to March 2020, using the following keywords in separate and in combination: “myocarditis,” “cardiac magnetic resonance,” “magnetic resonance,” “CMR,” “MR,” and “MRI.” Additionally, references from identified studies were manually searched. Two independent authors first reviewed titles and abstracts to retrieve relevant articles, and then read the full texts to identify the included studies. Discrepancies were resolved by consensus.

### 2.2 Inclusion and exclusion criteria

Studies were considered eligible based on the following inclusion criteria: (1) cohort or case‐control studies evaluating the utility of CMR in adult patients with clinically suspected acute myocarditis; (2) using endomyocardial biopsy (EMB) or clinical criteria as the reference standard; (3) reporting T1 mapping, T2 mapping, or ECV; (4) using CMR on a 1.5T scanner, in order to minimize the inconsistency across the studies and given CMR at 1.5T was most commonly used in the literature; (5) providing the essential data to construct a 2 × 2 contingency table for calculation, namely true‐ and false‐positive values and true‐ and false‐negative values, if diagnostic yields were reported only; (6) complete analytic study written in English. If two or more studies were analyzing the same cohorts, the study with the most sufficient data was included. Studies that included patients with onset of symptoms >2 weeks before CMR were excluded. Studies enrolling complex patient cohorts without examining the outcomes of myocarditis patients alone were also excluded.

### 2.3 Data extraction

Data from each included study were independently extracted by two reviewers into a predefined Excel spreadsheet. The extracted data included: the first author's name, publication year, location, study design, sample size, patients selection criteria, patients baseline characteristic, reference standard, CMR imaging protocol, the interval from symptom onset to CMR, and CMR results on mapping techniques. Differences between reviewers were resolved by discussion.

### 2.4 Study quality assessment

The methodological quality of included studies was appraised using the QUADAS‐2 (Quality Assessment for Diagnostic Accuracy Studies) tool.^14^ The QUADAS‐2 tool consists of four domains, namely patient selection, index test, reference standard, and flow and timing. In each domain, the risk of bias and applicability of studies was rated as low, high, or unclear. The study assessment was completed by two independent researchers and consensus was achieved after discussion.

### 2.5 Statistical analysis

Continuous data including native T1 value, T2 relaxation time, and ECV were summarized using weighted mean differences (WMDs) and 95% CIs as their effect sizes, and a 95% CI excluding the point of no effect indicates statistical significance. The pooled sensitivity, specificity, and diagnostic odds ratio (DOR) with the accompanying 95% confidence intervals (CIs) were calculated to determine the diagnostic value of CMR for the detection of acute myocarditis. Summary receiver operating characteristic curves (SROCs) were generated to estimate the effect of sensitivity and specificity. The area under the curve (AUC) of the SROC was estimated to show the overall efficacy of a given CMR parameter, and a higher AUC value reflects better diagnostic performance.

Heterogeneity across studies was assessed using I^2^ statistic and an I^2^ > 50% was considered as significant heterogeneity. We chose a random‐effects model to estimate the overall effect if I^2^ > 50%; otherwise we used a fixed‐effects model. The threshold effect, which was a potential source of heterogeneity because of different cutoff values to define a positive or negative test result, was assessed by Spearman correlation coefficients. A strong positive correlation between sensitivity and [1 ‐ specificity] (*p* < 0.05) confirmed the evidence of a threshold effect. The least significant difference tests and Scheffe tests were used for comparing the diagnostic performance among the mapping techniques. A meta‐regression and subgroup analysis was performed to summarize the sensitivity and specificity based on reference standards and determine if different gold standards could affect the heterogeneity and overall diagnostic value. Additionally, tests of interaction were conducted to compare subgroups. We used STATA software version 15.0 to perform all statistical analyses.

## 3 RESULTS

### 3.1 Study identification

**FIGURE 1:**
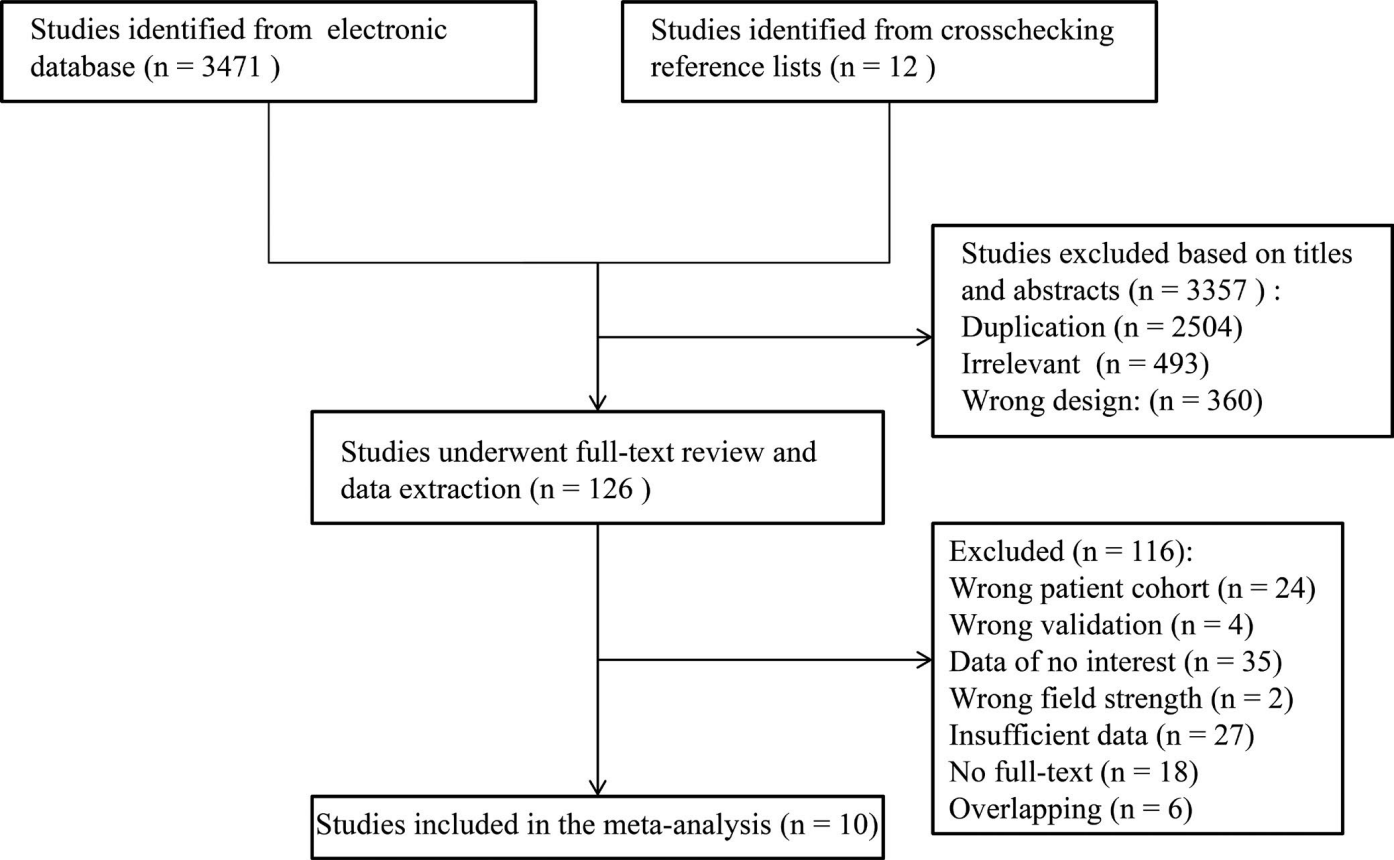
Flowchart of study identification

A total of 3483 articles were identified from the initial literature search. Of these, 3357 duplicated or irrelevant studies were excluded based on titles and abstracts, and the remaining 126 papers underwent full‐text review. Subsequent identification rejected 116 studies based on the eligibility criteria and 10 studies were finally included and used to meta‐analyze.^15^, ^16^, ^17^, ^18^, ^19^, ^20^, ^21^, ^22^, ^23^, ^24^ The flowchart of study selection was shown in Figure 1.

### 3.2 Study characteristics and quality

All the included studies were published between 2014 and 2019, with eight^16^, ^17^, ^18^, ^19^, ^20^, ^22^, ^23^, ^24^ of prospective design and two^15^, ^21^ of the retrospective design. Of these studies, only two studies^19^, ^20^ were conducted in multi‐center. The majority of studies were located in Germany. Totally, 666 participants were enrolled, with 400 in the myocarditis group and 266 in the control group. The mean age of the patients included varied from 24.5 to 54 years, with proportions of males ranging from 45% to 80%. Seven studies^15^, ^18^, ^19^, ^20^, ^21^, ^22^, ^24^ made the diagnosis of acute myocarditis based on clinical validation and three studies^16^, ^17^, ^23^ based on EMB findings. The study characteristics are summarized in Table 1.

**TABLE 1:** Study characteristics

First author	Year	Patient baseline characteristics (myocarditis group/control group)				CMR imaging protocol			Cutoff values			Reference standard
		*n*	Age	Male (%)	LVEF (%)	Scanner, vendor	T1 sequence	T2 sequence	T1 (ms)	T2 (ms)	ECV (%)	
Baeßler	2017	67/17	37 ± 14/36 ± 12	73/65	62 ± 7/65 ± 5	Achieva, Philips	N/A	GraSE	N/A	N/A	N/A	Clinical criteria
Baessler	2018	26/13	32 ± 14.8/35 ± 4.4	51/54	51 ± 5.9/56 ± 7.4	Intera CV, Philips	MOLLI 3(3)5	MESE	N/A	N/A	N/A	EMB
Baessler	2019	21/10	46 ± 16/47 ± 18	67/70	32 ± 18.5/27 ± 17	Intera CV, Philips	MOLLI 3(3)5	MESE	N/A	N/A	N/A	EMB
Dabir	2019	50/30	38 ± 16.3/36.9 ± 13.5	74/77	55.3 ± 9.4/61.6 ± 4.6	Ingenia, Philips	MOLLI 3(3)5	GraSE	>980	>54	>31	Clinical criteria
Ferreira	2014	60/50	41 ± 16/41 ± 13	75/74	64 ± 12/72 ± 6	Avanto, Siemens	ShMOLLI 5(1)1(1)1	N/A	≥990	N/A	N/A	Clinical criteria
Hinojar	2015	61/40	48 ± 17/45 ± 15	52/53	49 ± 15/61 ± 5	Achieva, Philips	MOLLI 3(3)3(3)5	N/A	>992	N/A	N/A	Clinical criteria
Huber	2018	20/20	35 ± 13/54 ± 18	80/45	53 ± 9/59 ± 4	Magnetom Aera, Siemens	MOLLI 3(3)5	GraSE	N/A	N/A	N/A	Clinical criteria
Luetkens	2016	34/50	44.9 ± 18.7/39.2 ± 17.2	50/60	55.5 ± 11.4/60.9 ± 3.6	Ingenia, Philips	MOLLI 3(3)3(3)5	GraSE	≥1000	≥55.9	≥28.8	Clinical criteria
Lurz	2016	43/18	40 ± 20	72	48 ± 19.3	Intera CV, Philips	MOLLI 3(3)5	MESE	>1058	>58.8	>32.6	EMB
von Knobelsdorff‐Brenkenhoff	2017	18/18	24.5 ± 11.1/26.5 ± 8.1	78/78	60 ± 4.4/61 ± 2.2	Magnetom Avanto, Siemens	MOLLI 5(3)3	T2‐SSFP	>980.7	>52.3	>24.1	Clinical criteria

Abbreviations: CMR, cardiovascular magnetic resonance; ECV, extracellular volume; EMB, endomyocardial biopsy; GraSE, Gradient Spin Echo; LVEF, left ventricular ejection fraction; MESE, multiecho spin‐echo; MOLLI, Modified Look‐Locker Inversion Recovery; N/A, not available; ShMOLLI, Shortened Modified Look‐Locker Inversion Recovery; T2‐SSFP, T2‐prepared steady‐state free precession.

The quality of the included studies was considered moderate and acceptable according to the QUADAS‐2 tool. In detail, all studies were assessed as low risk of bias in all domains with the exception of index tests, in which eight studies^17^, ^18^, ^19^, ^20^, ^21^, ^22^, ^23^, ^24^ were graded as unclear risk of bias since the blindness to the results of the reference standard being unreported, suggesting a possibility of applicability concerns. The summary of the critical appraisal is presented in Figure S1.

### 3.3 CMR characteristics

CMR results on native T1, T2 time, and ECV were reported in 9, 8, and 7 studies, respectively. After data synthesis, the pooled results showed that patients with acute myocarditis were associated with T1 prolongation (WMD: 69.15, 95% CI: 45.75–92.55, I^2^ = 90%), elevated T2 values (WMD: 5.42, 95% CI: 4.59–6.25, I^2^ = 18%), and increased ECV (WMD: 4.32, 95% CI: 3.10–5.52, I^2^ = 10%). All differences between groups were significant.

### 3.4 Native T1 mapping

**FIGURE 2:**
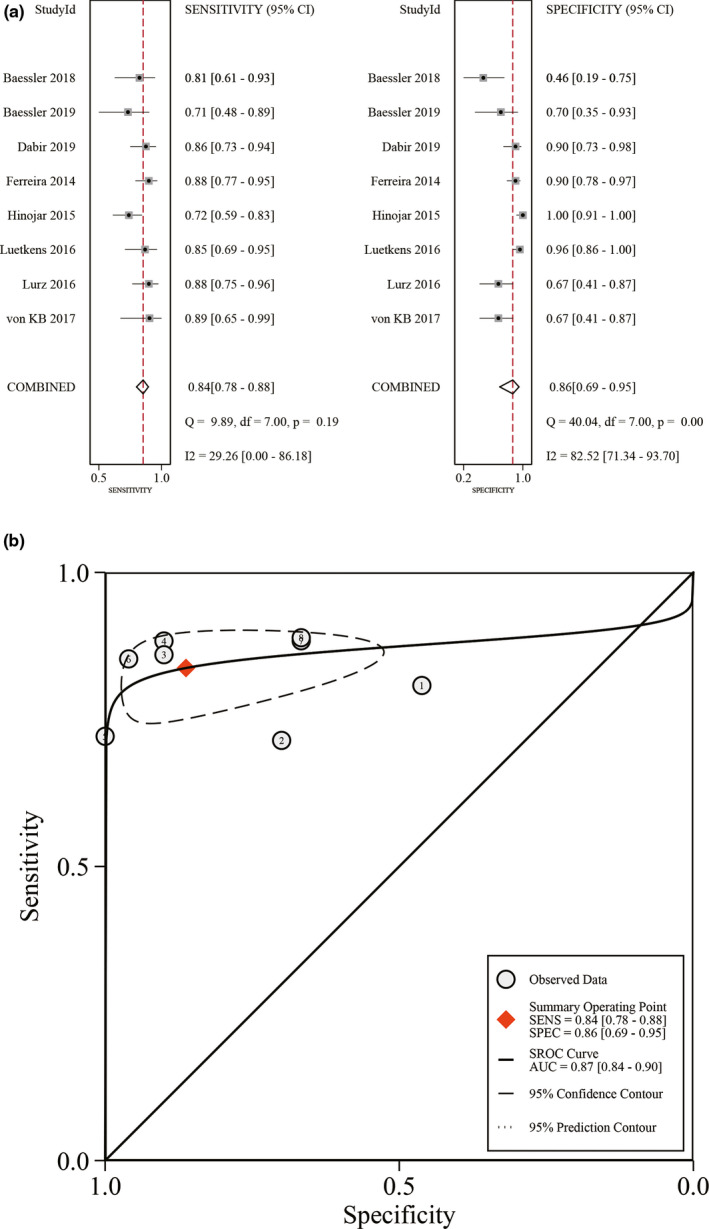
Diagnostic accuracy of native T1 mapping in the diagnosis of acute myocarditis. (A) Pooled sensitivity and specificity. (B) Summary receiver operating characteristic curve

The application of native T1 mapping for identifying acute myocarditis patients presented a pooled sensitivity of 0.84 (95% CI: 0.78–0.88, I^2^ = 29%), a pooled specificity of 0.86 (95% CI: 0.69–0.95, I^2^ = 82%) (Figure 2A). The pooled DOR was 32 (95% CI: 12–87). The AUC of SROC was 0.87 (95% CI: 0.84–0.90) (Figure 2B). The assessment of the Spearman correlation coefficient (−1.00; *p* = 1.00) showed no existence of a significant threshold effect.

### 3.5 T2 mapping

**FIGURE 3:**
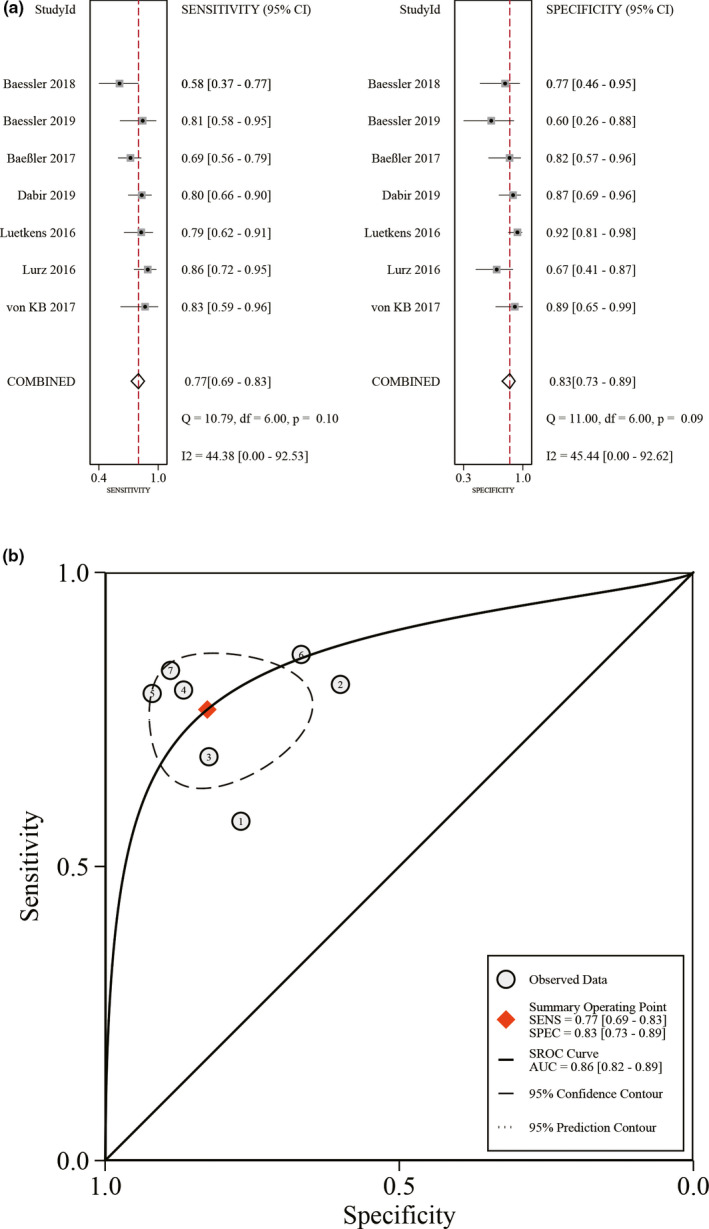
Diagnostic accuracy of T2 mapping in the diagnosis of acute myocarditis. (A) Pooled sensitivity and specificity. (B) Summary receiver operating characteristic curve

For the evaluation of the diagnostic performance of T2 mapping, the pooled sensitivity was 0.77 (95% CI: 0.69–0.83, I^2^ = 44%) and the pooled specificity was 0.83 (95% CI: 0.73–0.89, I^2^ = 45%) (Figure 3A). The pooled DOR was 16 (95% CI: 8–30). The AUC of SROC was 0.86 (95% CI: 0.82–0.89) (Figure 3B). The threshold effect was not present because the Spearman correlation coefficient was negative (−0.23) with *p* = 0.05.

### 3.6 ECV

**FIGURE 4:**
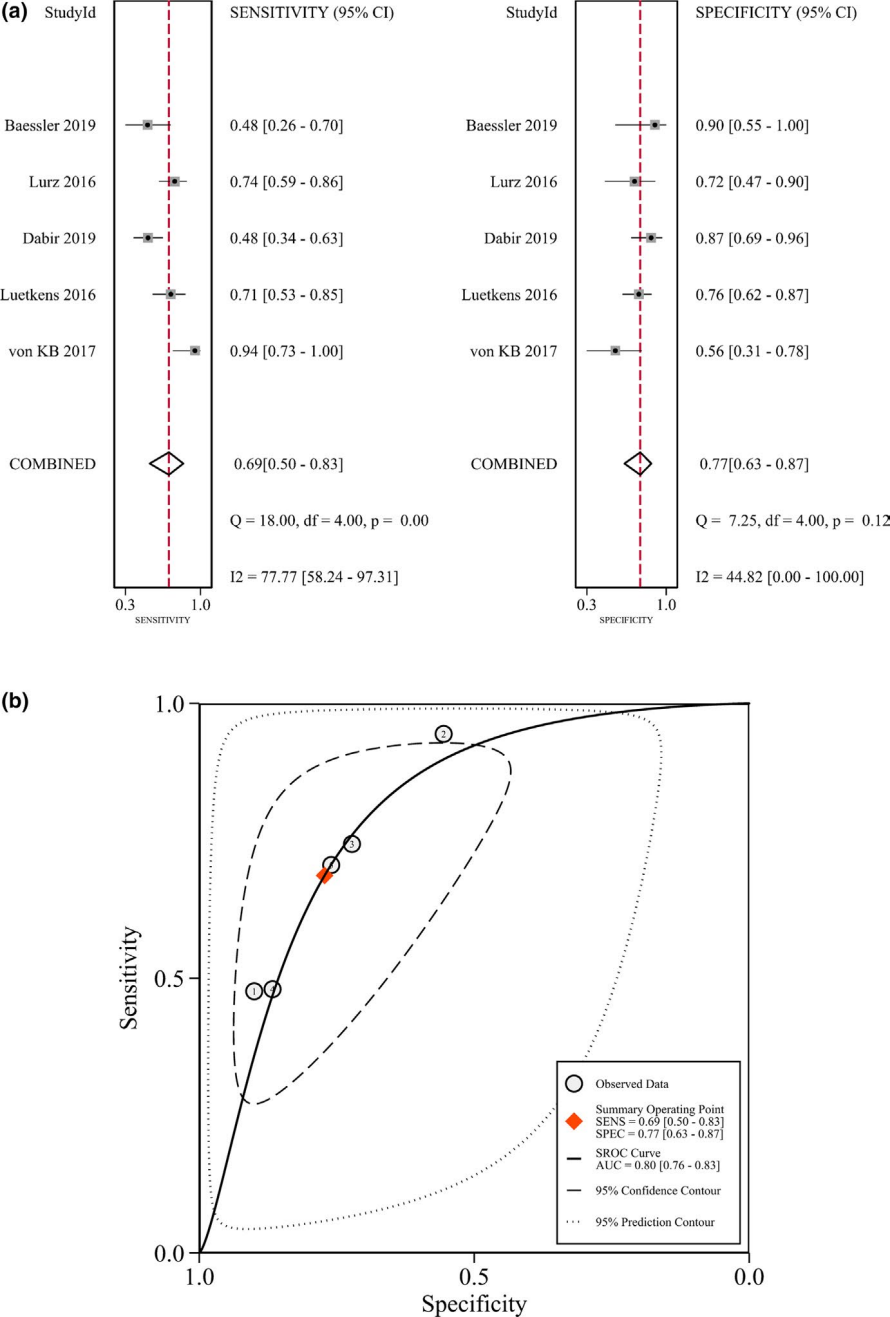
Diagnostic accuracy of extracellular volume in the diagnosis of acute myocarditis. (A) Pooled sensitivity and specificity. (B) Summary receiver operating characteristic curve

In the accuracy calculation for ECV mapping, the meta‐analysis generated a pooled sensitivity of 0.69 (95% CI: 0.50–0.83, I^2^ = 78%) and a pooled specificity of 0.77 (95% CI: 0.63–0.87, I^2^ = 45%) (Figure 4A). The pooled DOR was 7 (95% CI: 4–14). The AUC of SROC was 0.80 (95% CI: 0.76–0.83) (Figure 4B). Threshold analysis revealed a Spearman correlation coefficient of 0.60 (*p* = 0.36), indicating the absence of threshold effect in this group of studies.

### 3.7 Comparison of diagnostic performance

The least significant difference and Scheffe tests showed that there were no significant differences among the three mapping techniques regarding sensitivity, specificity, DOR, or SROC (*p* > 0.05).

### 3.8 Meta‐regression and subgroup analysis

A subgroup analysis, separating studies according to reference standards (clinical findings or EMB), was performed. The analysis revealed that reference standards had no significant effect on the diagnostic accuracy of each CMR mapping parameter or the heterogeneity across studies. (Table 2) When comparing the two subgroups, *P* value for tests of interaction showed that there were no significant differences, except that significantly lower specificities of T1 and T2 mapping were observed in EMB studies (*p* < 0.01).

**TABLE 2:** Results of meta‐regression and subgroup analyses based on reference standards

Mapping techniques	Reference standards	Studies	Sensitivity	*p* value	Specificity	*p* value
Native T1	Clinical criteria	5	0.85 (0.78–0.91)	0.06	0.92 (0.96–0.99)	0.07
	EMB	3	0.82 (0.73–0.92)		0.63 (0.36–0.89)	
T2	Clinical criteria	4	0.77 (0.68–0.85)	0.07	0.89 (0.83–0.95)	0.73
	EMB	3	0.76 (0.66–0.87)		0.68 (0.54–0.83)	
ECV	Clinical criteria	3	0.73 (0.52–0.93)	0.74	0.75 (0.60–0.90)	0.38
	EMB	2	0.63 (0.34–0.91)		0.81 (0.62–1.00)	

Abbreviations: ECV, extracellular volume; EMB, endomyocardial biopsy.

## 4 DISCUSSION

This meta‐analysis integrated the available evidence on CMR‐based quantitative T1 and T2 mapping in patients with acute myocarditis and demonstrated that native T1 and T2 including T1‐derived ECV were all significantly increased in the myocarditis group and could provide reliable diagnostic performance, with AUCs of above 0.8. However, both T1 and T2 specificity subsided in the setting of EMB‐proven myocarditis, indicating that other pathology with similar presentations might be often misdiagnosed by CMR mapping.

T1 and T2 values can be obtained directly and are independent of the variation in signal intensity, which allows for the quantitative and objective assessment of myocarditis. Native myocardial T1 relaxation time was significantly higher in patients with acute myocarditis compared to control subjects. The prolongation of T1 values is attributed to cellular edema, increased extracellular space and water, inflammation, and myocyte necrosis, all of which commonly occur in the early stage of myocarditis.^5^, ^25^ Then in the current study, this CMR approach yielded good diagnostic performance in patients suspected of having acute myocarditis as reflected by its AUC value of 0.87. Myocardial T2 relaxation time also has a close relationship with free tissue water content, thus making it a promising marker to detect the diseased myocardium.^22^, ^26^, ^27^ T2 values were significantly elevated in patients compared with controls according to our results, and T2 mapping had a comparable diagnostic yield to native T1 with an AUC of 0.86.

The ECV is estimated from pre‐ and post‐contrast T1, which may allow for extracellular volume space quantification and accurate myocardial fibrosis correlation.^28^ With statistical significance, an ECV elevation was detected in acute myocarditis. Following this result, ECV showed a good diagnostic potential to detect suspected acute myocarditis with an AUC of 0.80. However, the diagnostic performance might be hampered in the early course of the disease when the extent of interstitial edema might not suffice to allow for ECV elevation in diseased myocardium.^18^, ^22^, ^24^ In the same way, Dabir et al. yielded the lowest ECV sensitivity of 47% among the included studies with a short time to CMR of 3 days on average.^18^ Therefore, in the setting of suspected myocarditis with a short time interval between the onset of symptoms and CMR, the diagnosis employing exclusively extracellular information should be with caution.

As for the difference among T1, T2, and ECV, they have different diagnostic targets. While T1 is non‐specific for infiltration or edema, T2 is specific for edema, especially intracellular. Therefore, T2 quickly loses sensitivity after the acute phase, but is very specific for acute phase, T1 and ECV may stay positive even after acute inflammation. What is more, neither T1 nor ECV is specific for acute inflammation early after symptom onset. Pre‐existing myocardial injury may render patients more susceptible to inflammation and thus, T1 or ECV may have a higher prevalence in this population and not necessarily reflect acute disease. This may explain the observed lower sensitivity of T2 in comparison to T1 in this meta‐analysis. We know from previous experiences with CMR in myocarditis that the combination of CMR parameters might be better than single parameters.^6^ In this paper, the included study of von Knobelsdorff‐Brenkenhoff et al. showed a high diagnostic performance when combining native T1 and T2, with a sensitivity of 0.78 and a specificity of 0.94. Based on two parameters, the diagnosis of acute myocarditis is even more secure. However, this result was achieved from clinically proven myocarditis, and the evidence should be further investigated.

Having more stringent inclusion criteria and exclusively analyzing CMR mapping techniques at 1.5 T, the present study showed a similar diagnostic yield of both T1 and T2 mapping, as reported by two previous meta‐analyses.^10^, ^11^ However, considering the variability of reference standard may bias the results, subgroup analysis based on validation was conducted. Interestingly, when differentiating participants with positive findings at EMB from those with negative findings, we found a reduction in both specificities of T1 (0.63 vs. 0.92; *p* < 0.01) and T2 (0.68 vs. 0.89; *p* < 0.01) in the EMB‐based studies than those in clinically diagnosed studies. The main reason may be that the T1 and T2 mapping lack specificity for acute myocarditis as compared with other pathology with similar symptoms. Besides, Radunski et al.^29^ found T1 and T2 mapping were not sensitive enough to acute myocarditis when they included patients with subacute or chronic myocarditis (T1: 0.64; T2: 0.57). That is to say, our results confirm that native T1 and T2 may allow for a reliable distinction between injured and normal myocardium, but still struggle to discriminate between acute myocarditis and noninflammatory cardiomyopathy or myocarditis with a chronic presentation, which is of more clinical significance and deserves further evaluation in CMR studies. Perhaps, multiparametric imaging approach to acute myocarditis may yield superior diagnostic efficacy,^15^ to which attention should be played in future studies.

The present meta‐analysis represents more complete analysis for the diagnostic value of CMR‐based T1 and T2 mapping techniques than prior published meta‐analyses^10^, ^11^, ^12^ as this study included more recent studies and made an additional analysis based on the type of validation test. More importantly, we restricted the timing from the onset of symptoms to CMR assessment to ≤14 days in patients. The findings would help to provide more insight into the diagnostic value of T1 and T2 mapping and inform better guidelines for patients undergoing CMR for the suspicion of acute myocarditis. However, some limitations must be acknowledged. First, even though a meta‐regression analysis had been conducted to explore the source of heterogeneity, significant heterogeneity remained in T1 specificity and ECV sensitivity, which may be resulted from various population selection, patient baseline characteristics, observers' interpretation, or CMR acquisition protocols. Second, all the included CMR data are performed on a 1.5‐T scanner from only two vendors (Philips and Siemens), thus the results may not be generalizable to other field strengths or vendors. Third, the number of the included studies was still small, which limited us to conduct publication bias and draw a definite conclusion. Thus, more studies are needed to verify our results, especially in patients' cohorts with EMB‐proven myocarditis.

## 5 CONCLUSION

In conclusion, the currently available evidence shows that T1 and T2 mapping including ECV alone offer comparably good diagnostic performance for the detection of acute myocarditis. The reason for the observed mismatch with EMB findings should be further investigated.

## CONFLICTS OF INTERESTS

No conflicts of interest.

## AUTHOR CONTRIBUTION

Zhi Jia made contribution to the conception of the work, the interpretation of data, and revise the manuscript. Lihong Wang and Yanqing Jia contributed to the acquisition of data. Hong Zhao and Liwei Huo were responsible for the analysis of data. Lihong Wang and Binbin Zheng contributed to draft the work. All authors had approved the manuscript.

## Supporting information

Fig S1

## References

[1] Eckart RE , Scoville SL , Campbell CL , et al. Sudden death in young adults: a 25‐year review of autopsies in military recruits. Ann Intern Med. 2004;141(11):829. 15583223 10.7326/0003-4819-141-11-200412070-00005

[2] Felker GM , Thompson RE , Hare JM , et al. Underlying causes and long‐term survival in patients with initially unexplained cardiomyopathy. N Engl J Med. 2003;342(15):1077‐1084. 10.1056/NEJM200004133421502 10760308

[3] Felker GM , Hu W , Hare JM , et al. The spectrum of dilated cardiomyopathy. The Johns Hopkins experience with 1,278 patients. Medicine. 1999;78:270‐283. 10424207 10.1097/00005792-199907000-00005

[4] Caforio AL , Pankuweit S , Arbustini E , et al. Current state of knowledge on aetiology, diagnosis, management, and therapy of myocarditis: a position statement of the European Society of Cardiology Working Group on myocardial and pericardial diseases. Eur Heart J. 2013;34:2636‐2648. 23824828 10.1093/eurheartj/eht210

[5] Friedrich MG , Sechtem U , Schulz‐Menger J , et al. Cardiovascular magnetic resonance in myocarditis: a JACC white paper. J Am Coll Cardiol. 2009;53(17) :1475‐1487. 19389557 10.1016/j.jacc.2009.02.007 PMC2743893

[6] Ferreira VM , Schulz‐Menger J , Holmvang G , et al. Cardiovascular magnetic resonance in nonischemic myocardial inflammation: expert recommendations. J Am Coll Cardiol. 2018;72(24):3158‐3176. 30545455 10.1016/j.jacc.2018.09.072

[7] Diao KY , Yang ZG , Xu HY , et al. Histologic validation of myocardial fibrosis measured by T1 mapping: a systematic review and meta‐analysis. J Cardiovasc Magn Reson. 2016;18(1):92. 27955698 10.1186/s12968-016-0313-7 PMC5154013

[8] Fernandez‐Jimenez R , Sanchez‐Gonzalez J , Aguero J , et al. Fast T2 gradient‐spin‐echo (T2‐GraSE) mapping for myocardial edema quantification: first in vivo validation in a porcine model of ischemia/reperfusion. J Cardiovasc Magn Reson. 2015;17:92. 26538198 10.1186/s12968-015-0199-9 PMC4634909

[9] Klein C , Nekolla SG , Balbach T , et al. The influence of myocardial blood flow and volume of distribution on late Gd‐DTPA kinetics in ischemic heart failure. J Magn Reson Imaging. 2004;20(4):588‐594. 15390232 10.1002/jmri.20164

[10] Kotanidis CP , Bazmpani MA , Haidich AB , et al. Diagnostic accuracy of cardiovascular magnetic resonance in acute myocarditis: a systematic review and meta‐analysis. JACC Cardiovasc Imaging. 2018;11(11):1583‐1590. 29454761 10.1016/j.jcmg.2017.12.008

[11] Pan JA , Lee YJ , Salerno M . Diagnostic performance of extracellular volume, native T1, and T2 mapping versus Lake Louise criteria by cardiac magnetic resonance for detection of acute myocarditis: a meta‐analysis. Circ Cardiovasc Imaging. 2018;11(7):e007598. 30012826 10.1161/CIRCIMAGING.118.007598 PMC6192699

[12] Blissett S , Chocron Y , Kovacina B , et al. Diagnostic and prognostic value of cardiac magnetic resonance in acute myocarditis: a systematic review and meta‐analysis. Int J Cardiovasc Imaging. 2019;35(12):2221‐2229. 31388815 10.1007/s10554-019-01674-x

[13] Moher D , Liberati A , Tetzlaff J , et al. Preferred reporting items for systematic reviews and meta‐analyses: the PRISMA statement. J Clin Epidemiol. 2009;62(10):1006‐1012. 19631508 10.1016/j.jclinepi.2009.06.005

[14] Whiting PF , Rutjes AW , Westwood ME , et al. QUADAS‐2: a revised tool for the quality assessment of diagnostic accuracy studies. Ann Intern Med. 2011;155(8):529. 22007046 10.7326/0003-4819-155-8-201110180-00009

[15] Baeßler B , Treutlein M , Schaarschmidt F , et al. A novel multiparametric imaging approach to acute myocarditis using T2‐mapping and CMR feature tracking. J Cardiovasc Magn Reson. 2017;19(1):71. 28931401 10.1186/s12968-017-0387-x PMC5607501

[16] Baessler B , Luecke C , Julia Lurz J , et al. Cardiac MRI texture analysis of T1 and T2 maps in patients with infarctlike acute myocarditis. Radiol. 2018;289(2):357‐365. 10.1148/radiol.2018180411 30084736

[17] Baessler B , Luecke C , Lurz J , et al. Cardiac MRI and texture analysis of myocardial T1 and T2 maps in myocarditis with acute versus chronic symptoms of heart failure. Radiol. 2019;292(3):608‐617. 10.1148/radiol.2019190101 31361205

[18] Dabir D , Vollbrecht TM , Luetkens JA , et al. Multiparametric cardiovascular magnetic resonance imaging in acute myocarditis: a comparison of different measurement approaches. J Cardiovasc Magn Reson. 2019;21(1):54. 31462282 10.1186/s12968-019-0568-x PMC6714458

[19] Ferreira VM , Piechnik SK , Dall'Armellina E , et al. Native T1‐mapping detects the location, extent and patterns of acute myocarditis without the need for gadolinium contrast agents. J Cardiovasc Magn Reson. 2014;16(1):36. 24886708 10.1186/1532-429X-16-36 PMC4041901

[20] Hinojar R , Foote L , Arroyo Ucar E , et al. Native T1 in discrimination of acute and convalescent stages in patients with clinical diagnosis of myocarditis: a proposed diagnostic algorithm using CMR. JACC Cardiovasc Imaging. 2015;8(1):37‐46. 25499131 10.1016/j.jcmg.2014.07.016

[21] Huber AT , Bravetti M , Lamy J , et al. Non‐invasive differentiation of idiopathic inflammatory myopathy with cardiac involvement from acute viral myocarditis using cardiovascular magnetic resonance imaging T1 and T2 mapping. J Cardiovasc Magn Reson. 2018;20(1):11. 29429407 10.1186/s12968-018-0430-6 PMC5808400

[22] Luetkens JA , Homsi R , Sprinkart AM , et al. Incremental value of quantitative CMR including parametric mapping for the diagnosis of acute myocarditis. Eur Heart J Cardiovasc Imaging. 2016;17(2):154‐161. 26476398 10.1093/ehjci/jev246 PMC4882886

[23] Lurz P , Luecke C , Eitel I , et al. Comprehensive cardiac magnetic resonance imaging in patients with suspected myocarditis the MyoRacer‐trial. J Am Coll Cardiol. 2016;67(15):1800‐1811. 27081020 10.1016/j.jacc.2016.02.013

[24] von Knobelsdorff‐Brenkenhoff F , Schüler J , Dogangüzel S , et al. Detection and monitoring of acute myocarditis applying quantitative cardiovascular magnetic resonance. Circ Cardiovasc Imaging. 2017;10(2):e005242. 28213448 10.1161/CIRCIMAGING.116.005242

[25] Liu PP , Mason JW . Advances in the understanding of myocarditis. Circulation. 2011;104(9):1076‐1082. 10.1161/hc3401.095198 11524405

[26] Thavendiranathan P , Walls M , Giri S , et al. Improved detection of myocardial involvement in acute inflammatory cardiomyopathies using T2 mapping. Circ Cardiovasc Imaging. 2012;5(1):102‐110. 22038988 10.1161/CIRCIMAGING.111.967836 PMC3261300

[27] Bohnen S , Radunski UK , Lund GK , et al. Performance of T1 and T2 mapping cardiovascular magnetic resonance to detect active myocarditis in patients with recent‐onset heart failure. Circ Cardiovasc Imaging. 2015;8(6):e003073. 26015267 10.1161/CIRCIMAGING.114.003073

[28] White SK , Sado DM , Fontana M , et al. T1 mapping for myocardial extracellular volume measurement by CMR: bolus only versus primed infusion technique. JACC Cardiovasc Imaging. 2013;6(9):955‐962. 23582361 10.1016/j.jcmg.2013.01.011

[29] Radunski UK , Lund GK , Stehning C , et al. CMR in patients with severe myocarditis: diagnostic value of quantitative tissue markers including extracellular volume imaging. JACC Cardiovasc Imaging. 2014;7(7):667‐675. 24954462 10.1016/j.jcmg.2014.02.005

