# Supplementary figures and images for: Detection of acute myocarditis using T1 and T2 mapping cardiovascular magnetic resonance: A systematic review and meta‐analysis
Source: J Appl Clin Med Phys. 2021 Sep 4;22(10):239–48. doi: 10.1002/acm2.13365 (PMC8504590; doi:10.1002/acm2.13365)

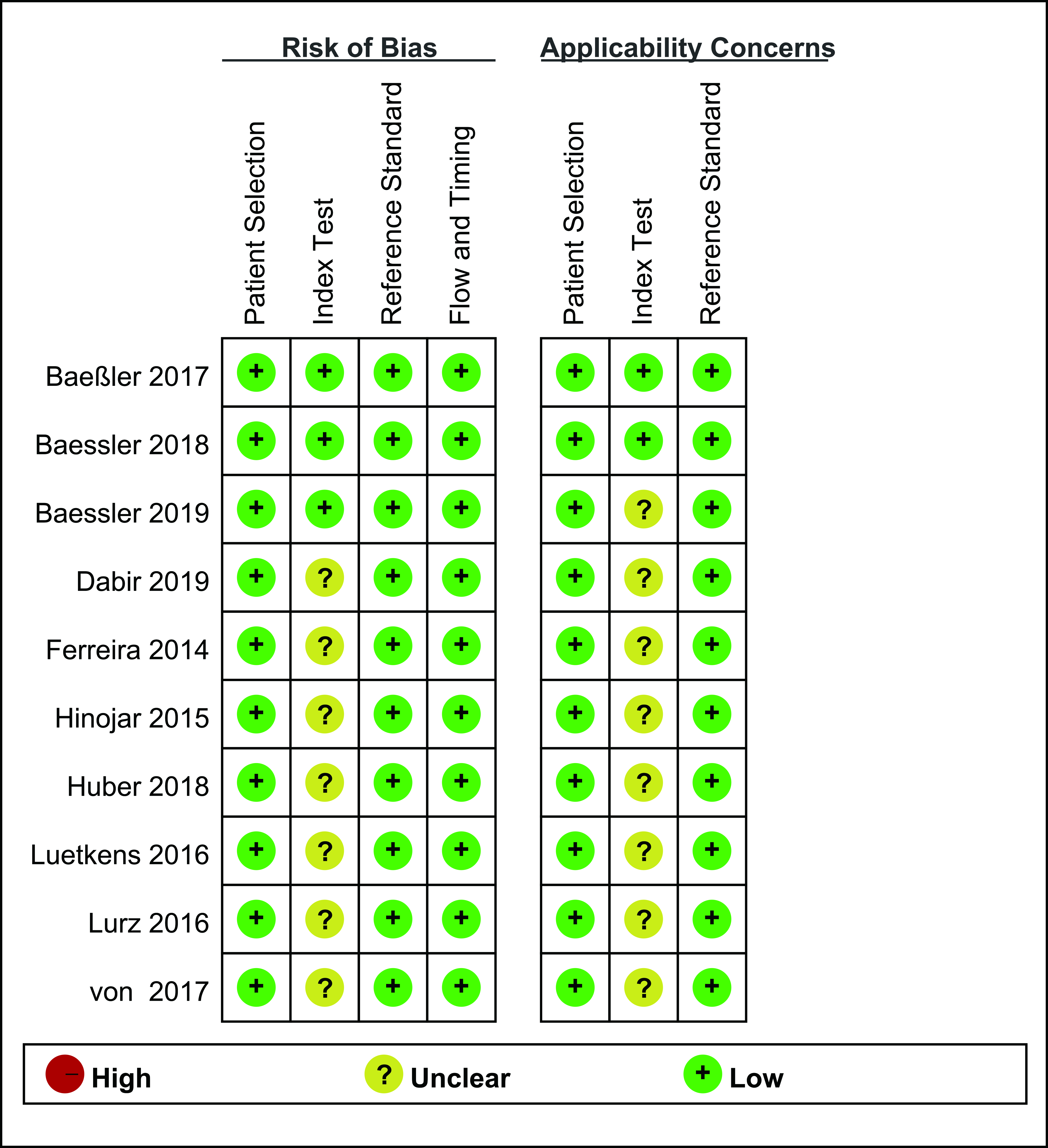

Supplement: Supplementary file 1 — Fig S1 [file ACM2-22-239-s001.tif]
